# Supplementary material for: Voltage Sensing in Bacterial Protein Translocation
Source: Biomolecules. 2020 Jan 3;10(1):78. doi: 10.3390/biom10010078 (PMC7023257; doi:10.3390/biom10010078)
Supplement: Supplementary file 1 [file biomolecules-10-00078-s001.pdf]

Supplementary Material

Voltage Sensing in Bacterial Protein Translocation

Denis G. Knyazev [1,‡], Roland Kuttner [1,‡], Ana-Nicoleta Bondar [2], Mirjam Zimmerman [1], Christine Siligan [1] and Peter Pohl [1,*]

1 Institute of Biophysics, Johannes Kepler University Linz, Gruberstr. 40, 4020 Linz, Austria; denis.knyazev@jku.at (D.G.K.); Rolandkuttner@gmx.at (R.K.); irjam_zimmermann@hotmail.com (M.Z.); Christine.Siligan@jku.at (C.S.)

2 Freie Universität Berlin, Department of Physics, Theoretical Molecular Biophysics Group, Arnimallee 14, D-14195 Berlin, Germany; nbondar@zedat.fu-berlin.de

‡ These authors contributed equally to this work

* Correspondence: Peter.Pohl@jku.at

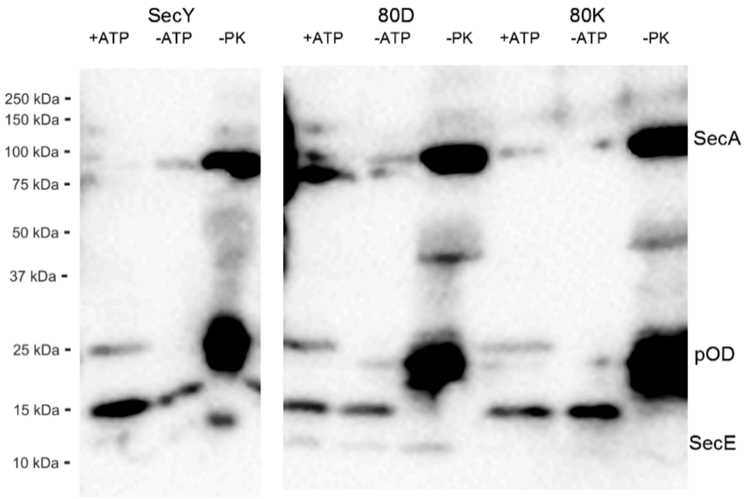

Figure S1: Translocation Assay. Western Blot detection with HRP - anti-His (Milenitec) Bands at 27 kDA show translocated proOmpA-DHFR in ATP positive samples for SecYEG "wild type" (single cysteine SecYA204C), and with mutations L80D, L80K.

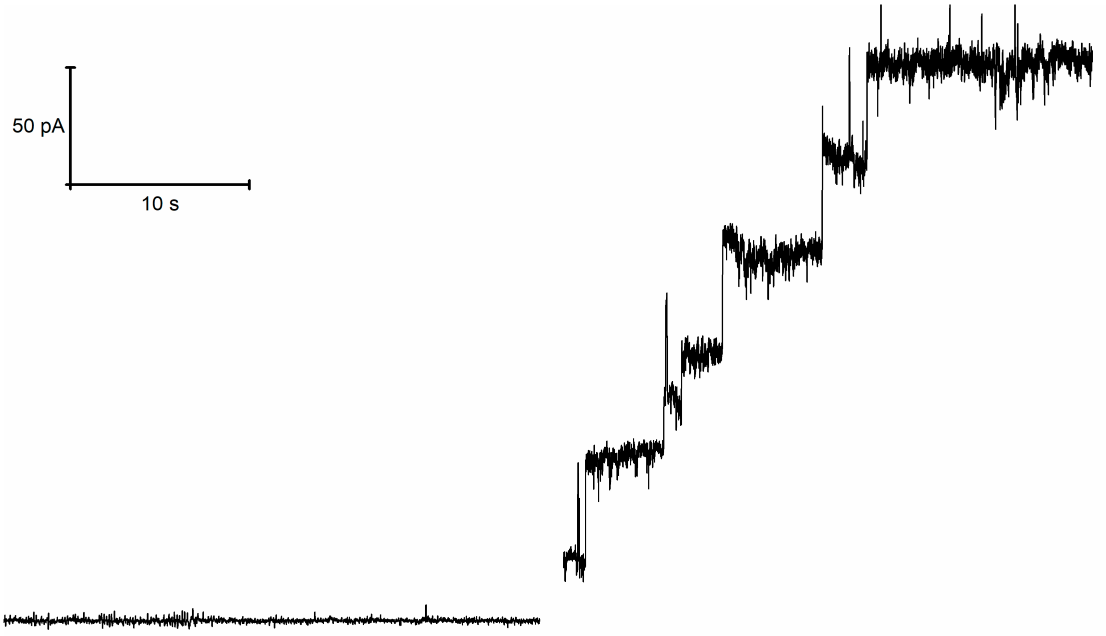

Figure S2. Controls for SecY(329C)EG. Left: The channels remained closed in the absence of the signal peptide from pOA. Application of φ = −85 mV did not elicit an increment in current. Right: Voltage triggered closings of SecYEG in complex with the signal peptide from pOA (in the absence of KTT). Application of much smaller φ values (= −85 mV) rendered the closing kinetics comparable to those observed under cross-linking conditions (Figure 2A,C, φ = −140 mV).

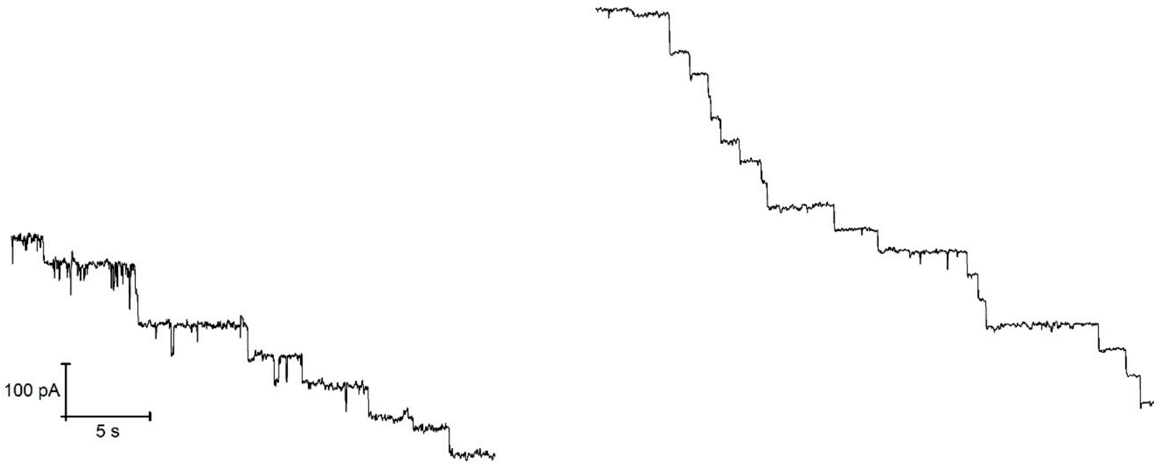

Figure S3. Voltage gated closings of (i) the SecYEG in complex with FtsQ-RNC (left panel) and (ii) the SecY(F67C)E(S120C)G mutant with the plug crosslinked to SecE by 1 mM KTT (right panel) at φ = +105 mV.
